# Emotional Exhaustion Scale (ECE): Psychometric Properties in a Sample of Portuguese University Students

**DOI:** 10.3390/ejihpe14040068

**Published:** 2024-04-17

**Authors:** Sílvia Ala, Francisco Ramos Campos, Inês Carvalho Relva

**Affiliations:** 1 Department of Social Sciences, Life and Public Health Polytechnic Institute of Bragança, School of Health, 5300-121 Bragança, Portugal; 2 Research Group on Neuroscience and Psychiatric Illnesses in Instituto de Investigation Sanitaria Galicia Sur, 36213 Pontevedra, Spain; 3 Department of Personality, Assessment and Psychological Treatments, Faculty of Psychology, University of Salamanca, 37005 Salamanca, Spain; frc@usal.es; 4 Department of Education and Psychology, University of Trás-os-Montes e Alto Douro, 5000-801 Vila Real, Portugal; irelva@utad.pt; 5 Research Center in Sports Sciences, Health Sciences and Human Development (CIDESD), University of Trás-os-Montes e Alto Douro, 5000-801 Vila Real, Portugal; 6 Centre for Research and Intervention in Education (CIIE), University of Porto, 4099-002 Porto, Portugal; 7 Centre of Psychology (CPUP), University of Porto, 4099-002 Porto, Portugal

**Keywords:** emotional exhaustion, university students, mental health, factor analysis

## Abstract

Academic emotional exhaustion is the first stage of academic burnout syndrome, and it is necessary to assess it and intervene early, as the consequences can lead to harmful effects on psychological well-being. The main objectives of this study were to explore the psychometric properties of the Emotional Exhaustion Scale (ECE); to assess the association with suffering and positive well-being, using the MHI-5 Inventory; and to provide information on its validity and to compare it with these constructs. A total sample of 526 university students (81% female and 19% male) aged between 17 and 62 (*M* = 21.42 years and *SD* = 5.78) took part in this study. Exploratory and confirmatory factor analyses were carried out. In the internal consistency assessment, the ECE was 0.89 and the MHI-5 was 0.81 (Cronbach’s alpha). Exploratory factor analysis was carried out with Varimax rotation and confirmatory analysis, obtaining the factor that explains 50.5% of the variance. The results indicated that the ECE has adequate psychometric properties for use with higher education students in Portugal. Its use by mental health professionals in higher education institutions could be very relevant for screening for emotional exhaustion and thus preventing possible serious pathologies.

## 1. Introduction

The 2030 Agenda for Sustainable Development, in Goal 4—Quality Education—sets the target that by 2030, the percentage of people aged between 25 and 34 with higher education should be at least 45%. Portugal is showing a very positive trend in most indicators, with a high increase in the number of young people aged between 25 and 34 who have completed higher education, from 33% in 2015 to 44% in 2021 [1]. However, the expansion of access to higher education is not keeping pace with the general performance of students, and some are at risk of failing and dropping out [2]. In 2020/2021, the number of students who dropped out one year after starting a higher education course grew by almost two percentage points in undergraduate courses: from 9.1% to 10.8% [3].

Dropping out of school is a complex [4] and multidimensional phenomenon. When initial expectations do not match reality or are not met, students can face frustration and difficulties in adapting to their new academic experiences [5], with an impact on well-being [6] and emotional, behavioral, cognitive and physiological dysregulation caused by stress and exhaustion that can seriously affect the performance of university students [7], leading to burnout.

Burnout syndrome is not listed in the 5th Edition of the Diagnostic and Statistical Manual of Mental Disorders [8]. However, given its global relevance and the effects it has on physical and mental health, it has been recognized. In 2022, the WHO, in the 11th edition of the International Classification of Diseases, began to recognize the syndrome and it was included in the chapter on problems associated with employment or unemployment, receiving the code “QD85” [9].

In this context, the study of exhaustion/emotional fatigue, known as burnout, has become pertinent, leading to a progressive interest in validating measuring instruments, the best known and most cited of which is the Maslach Burnout Inventory (MBI), which assesses three areas: personal accomplishment, emotional exhaustion and depersonalization [10]. Thus, according to the theoretical model proposed by Maslach et al. [10], emotional exhaustion (caused by academic demands), depersonalization (attitude of indifference towards academic activities) and dissatisfaction with performance (awareness of inefficiency as a student) are the factors that make up academic burnout. However, the most prevalent among university students is emotional exhaustion, since the other two factors have not been observed significantly or recurrently [11,12]. Emotional exhaustion stems from a set of feelings related to excessive cognitive demands, including pressure, stress and chronic fatigue [13].

The authors in [12] state that burnout occurs in students in the final years of their degree, as well as in postgraduate students, with emotional exhaustion being the form in which it manifests itself. This led the authors to construct and validate the Emotional Exhaustion Scale (ECE), a specific scale that considers the last 12 months of the student’s life; the items are inspired by the MBI emotional exhaustion scale and include the concept of burnout. To this base were added items specially designed to assess the fatigue or emotional exhaustion of university students, derived from the level of demand and effort to get through their studies [12].

The psychometric properties of the ECE have been validated in different Latin American countries such as Mexico ([14] with α = 0.87; [15] with α = 0.90), Argentina ([16,17] with α = 0.87; Peru [11] with α = 0.91; and [18] with α = 0.90), Puerto Rico ([19] with α = 0.883), Chile ([20] with α = 0.89), Ecuador [21] and Brazil [22]. It is noteworthy that of these studies, only four [15,18,20] performed confirmatory factor analyses that supported the one-dimensional ECE model.

The study of psychometric properties with a Portuguese sample is extremely important. According to the literature, Portuguese students showed estimates of 55.4% for emotional exhaustion, 31.6% for depersonalization and 30.9% for academic efficacy [23]. Another study, released at the beginning of 2020 (pre-pandemic COVID-19 period), showed that half of the students attending higher education institutions were experiencing burnout. Emotional exhaustion was the most frequent indicator in the initial phase of burnout [24].

The aim of this study is to translate and cross-culturally validate the ECE from the original instrument by Ramos et al. [12]; estimate the psychometric properties of the scale; confirm the factor structure using structural equations; and assess construct validity using the Mental Health Inventory (MHI-5), which assesses emotional distress and psychological well-being. This study is pertinent because there is no validated instrument for the Portuguese population that only assesses the emotional exhaustion construct separately from burnout, which will allow us to develop a greater understanding of this problem. Emotional exhaustion can therefore be overlooked if it is not detected in good time, compromising both academic performance and students’ health and quality of life.

## 2. Materials and Methods

This is a quantitative, exploratory and comparative study [25], aimed at adapting and studying the psychometric properties of the scale. It is based on numerical data obtained through self-report instruments and is cross-sectional in nature, since the data were collected at a single point in time.

### 2.1. Sample

The sample for this study was made up of 526 students from Portuguese higher education institutions, 19% male and 81% female, aged between 17 and 62 (21.42 ± 5.79). As for the courses they attended, most were in the area of social and behavioral sciences (41%) and health (31%). Overall 42.6% were in the first year, 18.6% in the second year, 15.8% in the third year and 0.4% in the fourth year of a bachelor’s degree; 4.9% were in the first year and 3.2% in the second year of a master’s degree; and 0.2% were in postgraduate studies.

### 2.2. Instruments

A questionnaire was drawn up with sociodemographic data and the scales under study.

The Emotional Tiredness Scale (ECE) by Ramos et al. [12] consists of 10 items that measure emotional tiredness/exhaustion. The items are scored from 1 to 5 on a Likert scale. It is a one-dimensional scale with an acceptable level of internal consistency (alpha coefficient of 0.83) and a satisfactory homogeneity of the items (average correlation between items = 0.33), with a mean of 27.45 and a standard deviation of 6.31. 

The reduced version of the Mental Health Inventory (MHI-5, validated for the Portuguese population by Ribeiro [26]) was used. The MHI is part of the National Health Survey and is a mental health instrument recommended by the WHO for use in population health studies. It consists of five items on mental health and the results are classified using an indicator that measures the probable existence of psychological distress. It is a self-administered questionnaire that aims to assess mental health from a perspective that includes a positive dimension (psychological well-being, positive mental health status) and a negative dimension of mental health (psychological distress, psychological suffering). The cut-off point indicated by the author was used, after linear standardization (0 to 100), which states that MHI5 values < 60 correspond to moderate symptoms and MHI5 values < 52 correspond to severe symptoms of psychological distress [27]. The MHI-5 expresses the same results as the long version, with good psychometric properties (α = 0.80).

### 2.3. Procedures

Initially, it was necessary to obtain authorization for the research protocol. Authorization was requested from the Ethics and Data Protection Committee of the Polytechnic Institute of Bragança (Opinion No. 134/2023-453683). This study was carried out in accordance with the latest version of the Declaration of Helsinki.

The cultural validation of the EEC followed the Cultural Adaptation Process, which corresponds to linguistic and conceptual validation, in accordance with international guidelines. This process was carried out in four stages: translation, synthesizing, back-translation and spoken reflection. The translation was carried out by two independent bilingual Portuguese translators. The back-translation was carried out by two other bilingual translators with no prior knowledge of the original scale. The pre-test was applied to a sample similar to the accessible population, made up of 15 students, and also included oral reflection—thinking aloud. The protocol was completed online using the LimeSurvey platform (https://www.limesurvey.com/ (accessed on 19 December 2023)), taking an average of 15 min, respecting ethical principles such as confidentiality, voluntary participation and data anonymity.

### 2.4. Statistical Analysis

The analyses were carried out using the statistical program Statistical Package for Social Sciences—SPSS, version 28 (IBM Corp., Chicago, IL, USA, Released 2021). It comprised several stages: descriptive analysis for all variables, internal consistency analysis and exploratory and confirmatory factor analysis for the ECE; *p* value ≤ 0.001.

Once the assumptions of normality had been met, the statistical analysis was carried out. The degree of internal consistency of the dimensions under analysis was calculated using Cronbach’s alpha to determine whether the instruments used were reliable in relation to the sample. Exploratory factor analysis was carried out using principal component extraction and Varimax rotation. For the confirmatory factor analysis, the Statistical Package for Social Sciences—SPSS AMOS, version 26 [28]—was used. It included the Kaiser–Meyer–Olkin (KMO) measure of sampling adequacy and Barlett’s test of sphericity, measuring the approximate chi-square, degree of freedom (df) and significance (*p* ≤ 0.05). Regarding the factors, the discrepancy between the model and the actual sample data was measured using the Analysis of Variance Explained. As χ^2^ is sensitive to sample size and the probability of rejecting the hypothetical model increases as the sample size increases, the use of other indices is recommended [29]. Finally, Pearson’s correlation was used to assess the correlations between the variables, since the data had a normal distribution.

## 3. Results

Table 1 shows Cronbach’s alpha coefficients for each of the scales, as well as the measures of central tendency and the Kolmogorov–Smirnov test, which demonstrates the normality of the variables.

There is no defined cut-off point for the ECE scale, so we used descriptive statistics to describe the emotional exhaustion variable, with a mean of 30.12 (*SD* = 3.58), for a maximum score of 50; 36% of the students had high levels of emotional exhaustion, 33% had moderate levels and 30% had low levels.

In MHI-5, the results obtained are classified through an indicator of mental health that measures the probable existence of psychological suffering. The cutoff point indicated by the author was used, after linear standardization (0 to 100). In the sample, 40% of the participants had severe symptoms of psychological distress and 60% had moderate symptoms of psychological distress. These groups are defined by the MHI-5 score, based on a cut-off point in the score of equal to or less than 52 to identify the group in probable psychological distress [27].

### 3.1. Internal Consistency

The reliability analysis of Cronbach’s alpha was performed for the entire scale to calculate the reliability of the instrument. Table 2 shows the correlations between the items, with the total reliability of the instrument being 0.89, including all items. In addition, reliability analysis was performed for each of the ten items (Table 2).

### 3.2. Exploratory Factor Analysis

In the exploratory factor analysis performed to estimate the validity of the ECE, the method of factor extraction was principal components, with Varimax orthogonal rotation, which assumes independence between the factors found. The results of the Bartlett sphericity test are significant (χ^2^ = 2384.36; Gl = 45; *p* = 0.001). The Kaiser–Meyer–Olkin measure verified the adequacy of the sample for the factorial analysis (KMO = 0.897), and the values of the anti-image matrix were greater than 0.30, the minimum acceptable value of practical significance [30]. These results indicate that the necessary conditions were met to proceed with the factor analysis.

Consistent with the original scale [12], a single factor with an eigenvalue greater than one was extracted, which explained 50.5% of the variance, and the scree plot showed a component positioned before the inflection; the previous result tended to be confirmed.

### 3.3. Confirmatory Factor Analysis

**Figure 1:**
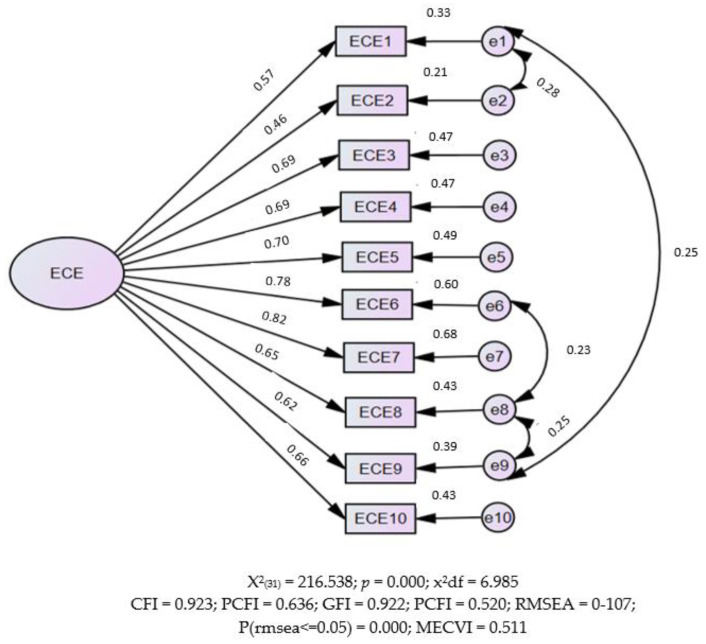
Confirmatory factor analysis of the modified model (n = 521).

In the CFA, the unifactorial model of the original ECE adjusted to a sample of 526 university students revealed a poor quality of fit. Table 3 shows the quality results of the fitting of the hypothetical model, with values below acceptable, suggesting that improvements in the model should be made. A more objective test (although affected by sample size) can be given by eliminating five outlier observations (square distance of Mahalanobis—D2). Analyzing the results of the post hoc analyses, significant covariances were observed between errors that were not predicted in the initial model. These covariances are found in item 1 (between e1 and e2, and e1 and e9), item 6 (between e6 and e8) and item 8 (between e8 and e9). Due to these unforeseen covariances, it was decided to perform the re-specification of the model. Figure 1 presents the new model tested, already with the coefficients estimated by the confirmatory analysis. According to the modification indices correlated with the measurement errors, it was possible to obtain significant parameters, and the indicators of the model show a good quality of fit (Table 3). Through the standardized coefficients of standardized factorial loads and the multiple correlation coefficients squared, R2, of the emotional exhaustion scale presented by a one-dimensional structure, we observed values of standardized factorial loads between 0.46 and 0.82 and a variability rate for the item explained by the emotional fatigue factor of 21% to 68%, as represented graphically in Figure 1.

It is observed, therefore, that, when compared to the saturated model (i.e., the first model), the second model has good results for fit quality, with CFI (0.92) and TLI (0.898) and the chi-square test (χ^2^ = 216.538, *p* < 0.000, χ^2^/Gl = 6 MEC. (0.5 vs. 0.7); however, the RMSEA (0.107) is above the recommended [29]. The composite reliability was revealed to be 0.549, and the mean extracted variance (MEV) of 0.45, an indicator of convergent validity, was revealed to be close to the recommended value (0.50), thus concluding that there is divergent validity. In this sense, the instrument seems to be adequate in this sample, so it can be a valuable resource for the study of emotional exhaustion in university students.

### 3.4. Validity

For the evidence of the discriminant and divergent validity of the EEC, it was analyzed in correlation with another variable subject to analysis with different psychological constructs, for which the Mental Health Inventory (MHI-5) was considered. Based on the objective of analyzing the associations between the dimensions under study, correlational analyses were performed between the different variables. Pearson correlations were performed, and they can assume a value between −1 and 1. This coefficient allows the association between variables to be determined, as well as the strength and intensity of this same association. They may be positive or negative, and present a low, moderate or strong degree of association [31]. According to Cohen [32], the correlation is weak when *r* = 0.10 to 0.29 or *r* = −0.10 to −0.29; moderate when *r* = 0.30 to 0.49 or *r* = −0.30 to −0.49, and strong when *r* = 0.50 to 1.0 or *r* = −0.50 to −1.0.

The correlation matrix between the ECE, the two factors and the total MHI-5 scale indicates positive, negative and significant correlations (Table 4), in which an increase or decrease in the indices of a variable corresponds to an increase or decrease in the variable with which it correlates. It is verified that emotional exhaustion presents a positive correlation with the average self-perception of mental health (*r* = 0.332, *p* = 0.001) and a strong correlation with psychological distress (*r* = 0.509, *p* = 0.001). On the other hand, it correlates negatively on average with psychological well-being (*r* = −0.340, *p* = 0.001). Thus, it appears that higher levels of emotional exhaustion are associated with higher levels of self-perception of mental health and psychological disorders (and vice versa). And although higher levels of emotional exhaustion will be associated with lower levels of psychological well-being (and vice versa), as the values of the correlations obtained are of medium intensity, it means that there is not much overlap between emotional exhaustion and the other dimensions (self-perception of mental health, distress and psychological well-being) assuming that the ECE items are an adequate measure of the construct to quantify emotional exhaustion.

## 4. Discussion

The objective of this study was to perform the translation and cross-cultural validation of the ECE from the original instrument of Ramos et al. [12]; the psychometric properties were analyzed and the factorial structure was confirmed through a confirmatory technique of data analysis. Using the same one-dimensional empirical structure, corroborated by the theoretical structure proposed by the authors in [12], we evaluated the construct validity through the Mental Health Inventory (MHI-5), which evaluates the self-perception of mental health, with the dimensions of psychological suffering/emotional difficulties and psychological well-being in Portuguese university students. 

The results were obtained in a sample of 526 higher education students, mostly female (81%), with a minimum age of 17 years and a maximum of 62 years (*M* = 21.42 ± 5.79), for the total sample. Regarding emotional exhaustion, the average is 30.12 (*SD* = 3.58), for a maximum score of 50. In the analysis of the level of emotional exhaustion, 36% of the students had high levels of tiredness/emotional exhaustion, 33% had moderate levels and 30% had low levels. This indicates that students were characterized by a set of somatic symptoms (tension, insomnia and headaches), as well as psychological (anxiety, stress, tension and frustration), which led to a decrease in energy to carry out academic activities and deal with university life.

Regarding mental health, 40% of the students in our sample show levels of psychological distress and a lower perception of mental health, with an absence of psychological well-being, while 60% had a positive perception of mental health associated with psychological well-being. Psychological distress is a long-lasting and current problem that affects university students globally and is related to a decrease in academic performance and results, failure in academic obligations and even dropping out of university [33].

When comparing the results of this study with those of the original scale [12], we can see that the variance explained in the EFA is higher than that obtained by the authors of the ECE, 40%; in the sample of our study, the variance explained was 50.50. This difference can be explained by the characteristics of the sample and the country of application. The existence of other studies that validated the instrument in Portugal is unknown, which limits the comparison of the results. 

The comparison of the internal structure of the ECE through exploratory and confirmatory factor analysis provides evidence on the one-dimensionality underlying the items of the scale. The studies of reliability and validity through exploratory and confirmatory factor analysis lead us to affirm that the ECE is a valid and reliable instrument, adapted to the study of the emotional exhaustion of Portuguese students in higher education.

It is observed that in the re-engineered model, there was a significant improvement in the adjustment indices of the model, in relation to the data obtained in the first model tested. The difference in χ^2^ values was 126.766 points, which is highly significant (given GL = 4). In addition, all other indices improved. This modification occurred due to the real existence of a correlation between the errors of the items (reason for the re-specification of the model) that was not initially predicted. These correlations have a theoretical justification: the items that have covariances with each other are related to the effects of mental fatigue/academic burnout on the psychological health of students. The covariance between the errors of items 1 and 2; 1 and 9; 6 and 8; and 8 and 9 may occur due to content similarity between these pairs. As can be observed in Table 1, the contents of items 1, 6, 8 and 9 are similar, since they deal with concepts related to the basic responses to stress. Probably, the notions of tension; tiredness; and lack of energy and effort, for respondents, are highly similar or are part of the same category, that is, responses related to the demands of work, presumably as a way of dealing with overload [34]. Perhaps this specific category is a peripheral indicator of the concept of mental exhaustion, yet this category seems to have a conceptual composition linked to other theoretical notions in psychology, such as self-referent phenomena (self-efficacy and self-concept). This covariance is due to this conceptual overlap.

Thus, the re-specification of the model was carried out in accordance with recommendations from the theoretical justification for its adoption [35]. The values of the model determination coefficients ranged from 0.46 to 0.82 and the item variability rate explained by the emotional fatigue factor from 21% to 68%, as can be seen in Figure 1. This indicates that the latent variable adequately predicts the variation in the observed variables. These estimated and high regression coefficients are also significant. All estimated variances, both those associated with measurement errors and those related to the latent variable, have statistical significance. This indicates a relationship with the items, which are relevant, and that the theory related to EC is present in them, as it is measured through personality, satisfaction with studies and psychological health [12].

However, the RMSEA presented a high magnitude, which was also found in other psychometric studies with the ECE [15,18,20]. The RMSEA must be less than 0.08 [29,36], although values equal to or less than 0.10 are allowed [37]. In other studies [15,18], it also exceeded the recommended acceptance limit (RMSEA = 0.12 and RMSEA = 0.10). However, it is not problematic, since the CFI is acceptable even in the presence of a high RMSEA [38].

The indicators of the adequacy of the re-specification model are quite satisfactory. However, it is still possible to improve the scale and, consequently, improve the adequacy indices. The items that presented covariances can be suppressed and new indicators of the events measured by these items can be formulated. In this way, one can end the redundancy observed in them, enabling the improvement in the adequacy of the model. In general, it can be said that the latent structure of the construct was confirmed and that the results are encouraging for the improvement in the measure by correcting the gaps indicated by the confirmatory factor analysis.

Regarding the internal consistency in our study (α = 0.89), it is comparable with other studies, which varied between 0.83 and 0.90 [12,14,15,16,18,20], demonstrating good internal consistency in the process of translation and validation of the ECE into Portuguese, being even superior to the report in the original study [12] (α = 0.83).

The results obtained in the analysis of associations between the dimensions under study indicate that emotional exhaustion has, in this sample, a mean positive correlation with self-perception of mental health (0.33) and a strong correlation with psychological distress—anxiety, depression and loss of emotional/behavioral control (0.51)—and a mean negative correlation with the perception of psychological well-being—positive affect and emotional ties (−0.34). Thus, with the use of the Pearson test, it is possible to affirm that higher levels of emotional exhaustion are associated with higher levels of self-perception of mental health and psychological distress (and vice versa); however, in relation to good psychological well-being, there is an inverse effect because, when one increases, the other decreases (and vice versa).

Emotional fatigue is the first experience that university students face, due to the demands of the academic environment. However, if they are unable to cope with it for a long period of time, their ability to carry out activities begins to be affected, altering their self-perception of mental health and psychological distress and their persistence and effort to make the most appropriate decisions to solve tasks; without fatigue, the appropriate confrontation of these demands will reinforce psychological well-being.

This study is not without limitations. It was a convenience sample and is not representative of Portuguese university students. Regarding sex, the sample was not homogeneous between males and females (19% and 81%, respectively), with females being under-represented. This can be explained by the fact that the study areas most represented in our sample were the health, social and behavioral sciences. According to data from 2021 [39], in terms of both enrolment and completion of higher education, females outnumber males in all areas, except for services, engineering, manufacturing and construction, and information and communication technologies (ICT). The data collection measure was self-reported, and participants may have a more positive view of themselves, influenced by social desirability.

In future research, the authors consider that studies on emotional fatigue as one of the factors that make up burnout should be continued and should focus on the design, application and evaluation of the effectiveness of prevention and intervention programs on the phenomenon evaluated, examining the relationship of this variable with sociodemographic characteristics and other constructs to see their interaction, such as anxiety, stress and depression, phenomena that occur frequently among university students.

## 5. Conclusions

This study describes the psychometric properties of the Emotional Exhaustion Scale in a sample of Portuguese university students. The results support the reliability and validity related to the construct of emotional exhaustion measured by the ECE. The unifactorial structure of the ECE was confirmed, with significant coefficients of medium and high magnitude of the ECE, showing the validity of the scale. This can be stated even though there was an indicated re-specification of the model due to the covariances not initially identified. However, the ECE has more evidence of validity that supports its use as an evaluation tool for the initial phase of academic burnout in university students. To better understand the mental health of Portuguese university students, this instrument should be applied to other samples and correlated with other phenomena, such as stress, anxiety and depression. It should be noted that the existing literature is not based on the use of an instrument to exclusively measure emotional exhaustion in this population. The results help in the description of phenomena related to emotional exhaustion, which is the dimension that stands out in academic burnout. It occurs when academic requirements exceed a tolerable level and lead to energy depletion. Students who are emotionally tired feel psychologically and emotionally drained. In addition to this empirical result, the theoretical reflection on the different events of emotional results helps to better describe this phenomenon.

In conclusion, the ECE, given its brevity and ease of understanding, can be used in clinical offices to support university students as a measure to detect early indicators of emotional exhaustion, and can help to outline interventions, prevention practices and awareness raising to stimulate the involvement of the academic community in the recognition and signaling of risk situations.

## Figures and Tables

**Table 1:** Descriptive statistics and normality tests.

Variable	Median	Means	*SD*	Kolmogorov–Smirnov	Cronbach’s Alpha
Emotional Exhaustion (ECE)	31	30.12	3.58	0.054; *p* = 0.001	0.89
MHI-5 Total ^a^	19	18.42	0.186	0.067; *p* = 0.000	0.81
Psychological distress	12	11.67	0.129	0.133; *p* = 0.000	0.81
Psychological well-being	6	6.75	0.083	0.178; *p* = 0.000	0.73

Note: *SD* = standard deviation. ^a^ Scale with inverted items.

**Table 2:** Item–total correlation and Cronbach’s alpha if item is excluded.

Items	Corrected Item–Total Correlation	Cronbach’s Alpha if Item is Excluded
1	0.607	0.877
2	0.456	0.888
3	0.610	0.877
4	0.659	0.873
5	0.626	0.876
6	0.708	0.871
7	0.748	0.867
8	0.616	0.876
9	0.608	0.877
10	0.613	0.877

**Table 3:** Adequacy indices of the models in the sample (n = 521).

				Absolute Fit Indices			Indices of Adjustment of Increment			
	χ^2^	df	X^2^/df	GFI	AGFI	RMR	RMSEA	NFI	TLI	CFI
Initial model	343.304	35	9.808	0.877	0.806	0.089	0.130	0.857	0.832	0.869
Adjusted model	216.538	31	6.985	0.922	0.861	0.067	0.107	0.912	0.888	0.923

Note: χ^2^: Chi-Square; df: degrees of freedom; GFI = Goodness of Fit Index; RMR = Root Mean Square Residual; RMSEA = Root Mean Square Error of Approximation; NFI = Normed Fit Index; TLI = Tucker–Lewis Index; CFI = Comparative Fit Index.

**Table 4:** Pearson correlation matrix between ACE and MHI 5.

	ECE	MHI-5	MHI_Distress	MHI_Psychological Well-Being
ECE	-			
MHI-5	0.332 **	-		
MHI_Distress	0.509 **	0.769 **	-	
MHI_Psychological well-being	−0.340 **	0.159 **	−0.508 **	-

** *p* < 0.001.

## Data Availability

The analyzed data are available upon reasonable request.
